# A novel antimicrobial peptide found in *Pelophylax* *nigromaculatus*

**DOI:** 10.1186/s43141-022-00366-9

**Published:** 2022-05-23

**Authors:** Chengyu Lu, Lingling Liu, Chengbang Ma, Liuqing Di, Tianbao Chen

**Affiliations:** 1 Jiangsu Provincial TCM Engineering Technology Research Centre of Highly Efficient Drug Delivery System (DDS), Nanjing University of Chinese Medicine, Nanjing, China; 2 Natural Drug Discovery Group, Faculty of Medicine, Health and Life Sciences, School of Pharmacy, Queen’s University Belfast, Belfast, UK

**Keywords:** Nigrocin, Antimicrobial peptide, Drug resistance

## Abstract

**Background:**

Many active peptides have been found in frog skin secretions. In this paper, our research focused on *Pelophylax nigromaculatus* and found a broad-spectrum antimicrobial peptide Nigrocin-PN based on the molecular cloning technique. Thereafter, the “Rana box” function was briefly studied by two mutated peptides (Nigrocin-M1 and Nigrocin-M2). Furthermore, in vitro and in vivo assays were used to characterize the peptide’s biofunctions, and the peptide’s function in treating multidrug-resistant pathogens was also studied.

**Results:**

Nigrocin-PN not only displayed potent antimicrobial abilities in vitro but also significantly ameliorated pulmonary inflammation induced by *Klebsiella pneumoniae *in vivo. By comparing, leucine-substituted analogue Nigrocin-M1 only displayed bactericidal abilities towards gram-positive bacteria, while the shorter analogue Nigrocin-M2 lost this function. More strikingly, Nigrocin-PN exhibited synergistic effects with commonly used antibiotics; in vitro evolution experiments revealed that coadministration between Nigrocin-PN and ampicillin could delay *Staphylococcus aureus* antibiotic resistance acquisition. Kinetics and morphology studies indicate that antibacterial mechanisms involved membrane destruction. Furthermore, toxicities and anticancer abilities of these peptides were also studied; compared to two analogues, Nigrocin-PN showed mild haemolytic activity and indistinctive cytotoxicity towards normal cell lines HMEC-1 and HaCaT.

**Conclusions:**

A broad-spectrum antimicrobial peptide Nigrocin-PN was discovered from the skin secretion of *Pelophylax nigromaculatus*. Structurally, “Rana box” played a crucial role in reducing toxicities without compromising antibacterial abilities, and Nigrocin-PN could be a desired therapeutic candidate.

**Graphical abstract:**

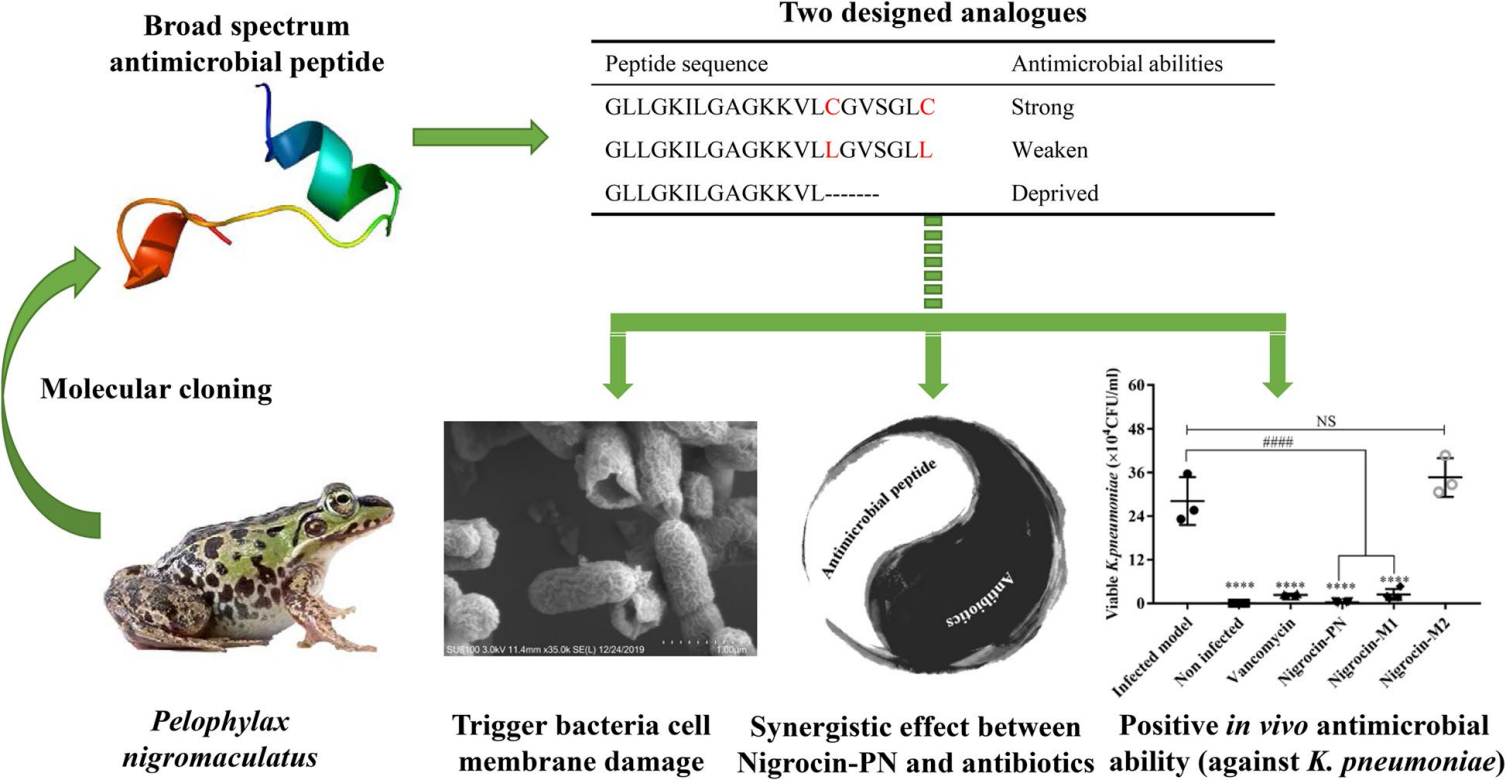

## Highlights

• For AMPs, disulphide bond can affect their biofunction and cytotoxicity.

• Frog skin secretion is a reservoir to delve valuable peptides.

• AMPs-antibiotics coadministration could be a strategy to delay drug resistance.

## Background

The rapid evolution and dissemination of antibiotic resistance among bacterial pathogens are outpacing the development of new antibiotics; some first-line antibiotics could not combat bacteria as effective as they used to be [1–3]. However, antimicrobial peptides (AMPs) provide an alternative; they elicit lower levels of resistance compared to conventional antibiotics and attack target bacteria by multimode of actions through membrane and intracellular targets. In general, there are five known pathways for antibiotics to work: (i) inhibition of bacterial cell wall biosynthesis, (ii) inhibition of protein synthesis, (iii) inhibition of nucleotides synthesis, (iv) cell membrane targeting, and (v) networking with antibiotic-induced stress response [4]. Nevertheless, bacteria could neutralise antibiotics by the following: (i) enzymatic degradation of antibacterial drugs, (ii) dodging the specific antibiotic target by altering proteins, and (iii) preventing influx and promoting efflux [5]. For AMPs, the possible bactericidal mechanism is membrane targeting; many modes of action such as “Barrel-stave model,” “Toroidal pore model,” and “Carpet model” were devised [6–8]. What is more, some host defence peptides (HDPs) could modulate the immune system, and the other AMPs even have intracellular targets and exert antibiotic-like functions [9–11]. These novel bactericidal mechanisms could inspire the development of next-generation antimicrobial agents.

Besides targeting to planktonic cells, it is also meaningful to highlight positive effects of AMPs to eradicate biofilms. Biofilms produced by pathogens are a great threat which not only provide shelters for bacteria but also make contributions to their dissemination. Several AMPs have been identified to inhibit initial attachment of planktonic bacterial cells on surfaces, promote disassembly of biofilm structures, and disrupt the stringent stress response [12]. However, the exact underlying mechanisms are complex and not fully understood. Research into antibiofilm can broaden the application of AMPs not only in medical use but also in material surface process.

The bioactivities of AMPs largely depend on their structures. Most AMPs carry positive charges ranging from + 2 to +13 [13], and some references also reported that the contents of α-helix and β-sheet can affect their bio-ability in a big scale [6, 7]. Furthermore, for the Nigrocin-2 family, the effect of “Rana Box” motif was quite ambiguous, a study on brevinine 1E revealed although this motif could stabilise the α-helix, it could not strengthen antimicrobial potency, while the elimination of disulphide bond in Nigrocin-HL significantly reduced its antibacterial ability [14, 15]. Thus, it is significant to probe the role of “Rana box” structure in Nigrocins.

Here, the isolation and identification of a novel AMP named Nigrocin-PN from the skin secretion of *Pelophylax nigromaculatus* were reported. The positive functions of “Rana box” were studied by employing two modified analogues: Nigrocin-M1 and Nigrocin-M2. Results revealed that the disulphide bridge was pivotal for reducing toxicities and maintaining bio-abilities. Nigrocin-PN showed prominent antibacterial abilities both in vitro and in vivo. Coadministration study indicated the synergistic effects between Nigrocin-PN and antibiotics such as ampicillin to combat *S. aureus*, etc. Moreover, the combination of Nigrocin-PN and ampicillin could delay the acquisition of resistance compared to using ampicillin independently.

## Methods

### Specimen biodata and secretion harvesting

Specimens of the dark-spotted frog, *Pelophylax nigromaculatus* (*n* = 3), were captured in Fujian Province, China. Before secretion acquiring, adaptive growth was conducted to ensure frogs’ good conditions. To obtain the skin secretion, the dorsal skin was stimulated by platinum electrodes under mild conditions (< 20 s; 6 V DC; 4 ms pulse width; 50 Hz), assisted by a hand massage. The viscous foamy white secretion was harvested by rinsing with deionised water and immediately snap-frozen in liquid nitrogen. After being lyophilised, it was stored at − 20 °C for further analysis. All the procedures were subject to ethical approval and carried out under appropriate UK animal research personal and project licences.

### “Shotgun” cloning of cDNA encoding a peptide precursor

Molecular cloning was carried out to acquire the putative cDNA encoding peptide precursor as the previous description [16] with minor discrepancies. The 3′-RACE reactions employed a NUP (nested universal primer; 5′-AAGCAGTGGTATCAACGCAGAGT-3′) which was supplied with the kit and a sense primer DV-3 (5′-GAWYYAYYHRAGCCYAAADATG-3′; *W* = A/T, *Y* = C/T, *H* = A/C/T, *R* = A/G, *D* = A/G/T) which was designed based on the highly conserved 5′-untranslated regions of the cDNA from *Pelophylax nigromaculatus*. RACE products were cloned by employing a pGEM®-T Easy Vector (Promega, USA) after being subjected to purification. Finally, the DNA sequencing reaction was built, and an ABI 3100 automated capillary sequencer (Applied Biosystems, USA) was used to determine the nucleotide sequences.

### Peptide modification and secondary structure prediction

The purpose of peptide modification was mainly focused on the function of the so-called Rana box. And the elimination of “Rana box” was reported to weaken haemolysis with no compromise of its bactericidal abilities both in nigrocin-2 and brevinin families [14, 15]. To reassure the function of this motif in nigrocin-2 family, two peptides which named Nigrocin-M1 and Nigrocin-M2 were devised. In Nigrocin-M1 (GLLGKILGAGKKVLLGVSGLL), leucine was used to replace the original cysteine to circumvent the function of the disulphide bond. Nigrocin-M2 (GLLGKILGAGKKVL) was a truncated version of Nigrocin-PN, and the “Rana box” circle was deleted. The devised peptides were all synthesised by tribute peptide synthesiser (Protein Technologies, USA) [17], purified by HPLC (high-performance liquid chromatography), and verified by MALDI-TOF (Matrix-assisted laser desorption ionization time-of-flight mass spectrometry).

JASCO J-815 circular dichroism (CD) spectrometer (JASCO Inc., USA) was used to determine the peptides’ secondary structures. The method was detailed in the previous study [18]. A total of 50 µM of each peptide was dissolved in the environment of 10 mM ammonium acetate (NH_4_Ac)/water and the membrane-mimic solution 50/50 (v/v) 2,2,2-trifluoroethanol (TFE)/10 mM NH_4_Ac, respectively. Then, the prepared solutions were loaded in a 1-mm thickness quartz cuvette, being analysed under the scanning range of 190–250 nm at the speed of 100 nm/min. All processes were performed at room temperature, and the data were acquired from three repetitions. Finally, DichroWeb (http://dichroweb.cryst.bbk.ac.uk/html/home.shtml) was used to estimate the contents of different secondary structures [19, 20], and K2D was chosen as a method to interpret the CD data.

Furthermore, physicochemical properties of the peptides were predicted by peptide property calculator (https://pepcalc.com/) and HeliQuest (http://heliquest.ipmc.cnrs.fr/cgi-bin/ComputParamsV2.py).

### In vitro antimicrobial susceptibility assay

The antimicrobial activities of three synthesised peptides were tested against both planktonic organisms as well as sessile cells in biofilms. In the first case, different microbes belonging to gram-positive, gram-negative, and fungi were listed as follows: *S. aureus* (NCTC 10,788), *S. aureus* (ATCC 25,923), *Enterococcus faecalis* (NCTC 12,697), MRSA (NCTC 12,493), MRSA (B038 V1S1A), MRSA (B042 V2E1A), *Streptococcus constellatus* (B003 VISIT), *Escherichia coli* (NCTC 10,418), *K. pneumoniae* (ATCC 43,816), *K. pneumoniae* (ATCC 13,883), *Pseudomonas aeruginosa* (ATCC 27,853), *P. aeruginosa* (ATCC 9097), *P. aeruginosa* (B004 V2S2B), and *Candida albicans* (NYCY 1467); among these strains, MRSA (B038 V1S1A), MRSA (B042 V2E1A), *S. constellatus* (B003 VISIT), and *P. aeruginosa* (B004 V2S2B) were clinically derived. Initially, each microorganism was incubated in Mueller–Hinton broth (MHB) overnight; then, the microorganism was subcultured and diluted to reach the concentration of 1 × 10^6^ CFU/ml for bacteria and 5 × 10^5^ CFU/ml for *C. albicans*. The appropriate concentrations of peptide stock solutions were prepared and added to the 96-well plate, which contained diluted organisms to obtain the concentration range from 512 µM to 1 µM. After 24 h incubation, the absorbance in each well was measured by the Synergy HT plate reader (BioTek, USA) at 550 nm wavelength, and the MICs (minimal inhibitory concentration) were defined as the lowest concentration of peptides at which no apparent growth was detected [21]. From the clear wells, 10 µl of the culture solution was added to a Mueller–Hinton agar (MHA) plate and incubated for another 20 h to detect each peptide’s MBC (minimal bactericidal concentration, which was defined as the lowest concentrations that showed no evidence of colony).

In the latter case, *S. aureus* (NCTC 10,788) and MRSA (NCTC 12,493) were used as reference strains to test peptides’ ability against sessile cells. MBIC (minimal biofilm inhibitory concentration) and MBEC (minimal biofilm eradication concentration) were used as indicators to estimate peptides’ antibiofilm ability. The main processes were the same as mentioned in reference [16], except for some modifications. Instead of using the specialised peg plate, the normal 96-wells round-bottom plate was used. Crystal violet staining method was used to detect the biomass to facilitate quantification [22, 23].

### Morphology observation of bacteria

Specific morphological changes of *K. pneumoniae* were observed by SEM. Firstly, *K. pneumoniae* were cultured under 16 and 0 µM of Nigrocin-PN for 12 h. Then, the bacteria were obtained by centrifugal separation under 6000 × *g* for 10 min and further washed with PBS several times. Samples for SEM were fixed in 2.5% glutaraldehyde for 4 h at 4 °C, followed by one washing step with PBS. Then, a series of increasing concentrations of ethanol (30, 50, 70, 80, 90, 95, and 100%) were used to dehydrate the samples for 15 min each time. After being processed by critical drying and coated with gold sputter, samples were examined by SEM [24].

### Time-killing assay

*S. aureus* (NCTC 10,788) and *E. coli* (NCTC 10,418) were used to detect the time-killing kinetics of Nigrocin-PN. The testing concentrations were set at 4 × , 2 × , and 1 × MIC. Bacteria and fungi were diluted with peptide-treated MHB to a concentration of 1 × 10^6^ CFU/ml. Ten microlitres of the growing bacteria and fungi were pipetted out at each specified time point from the incubator and diluted to calculate the colonies afterwards.

### Membrane permeability assay

The membrane permeability assay was performed as previously described with minor modifications [17, 25, 26]. The SYTOX Green was used as an indicator which can be monitored by Synergy HT plate reader under the conditions of excitation/emission: 485/528 nm. Here, *S. aureus* (NCTC 10,788), MRSA (NCTC 12,493), and *E. coli* (NCTC 10,418) were chosen as representatives of gram-positive and gram-negative strains to study the peptides’ putative antimicrobial mechanisms. Bacterial solutions were cultured, washed, and diluted [26] before transferring into a 96-well black plate, and 8 µM melittin in PBS was used as a positive control; twofold of MIC was chosen as the testing concentrations for Nigrocin-PN and Nigrocin-M1, while the concentration of Nigrocin-M2 was kept as the same as Nigrocin-PN for better comparison. During the operations, peptides and SYTOX Green were pre-added into the black plate before adding bacterial solutions. Once the bacteria were added, the plate was quickly placed into the reader for dynamic monitoring.

### Peptide cytotoxicity and anticancer evaluation

Defibrated horse blood cells (TCS Biosciences Ltd., Buckingham, UK), human keratinocyte cell line HaCaT, and human microvessel endothelial cell line HMEC-1 were used to assess peptides’ cytotoxicity. Non-small cell lung cancer H157, human prostate carcinoma PC-3, human glioblastoma astrocytoma U-251 MG, and human breast cancer MCF-7 were used to evaluate peptides’ antitumor ability.

In the haemolysis assay, 2% of horse erythrocytes were resuspended in PBS and incubated with each peptide at a concentration ranging from 512 to 1 µM. One percent of TritonX-100 (Sigma Aldrich, USA) was employed as a positive control. After 2 h of incubation at 37 °C, each peptide was centrifuged at 1000 × *g*, and 200 µl supernatant was pipetted out and measured under 550 nm.

MTT method was used to evaluate cell viability. The detailed method was described in reference [26], and mediums used to culture different cell lines were based on the cell product instructions. Briefly, A series of peptide concentrations ranging from 128 to 1 µM were employed to treat starved cells for 24 h, then 10 µl MTT (5 mg/ml) was added to each well and cultured for another 4 h, and the produced formazan was dissolved by 100 µl DMSO. The absorbance of the coloured solution was measured under 570 nm using the Synergy HT plate reader.

### Peptide and antibiotics coadministration study

*S. aureus* (NCTC 10,788), MRSA (NCTC 12,493), *E. coli* (NCTC 10,418), and *C. albicans* (NYCY 1467) were chosen to study the synergistic effect between Nigrocin-PN and several antibiotics (norfloxacin, ampicillin, vancomycin, and gentamicin). Checkerboard assay was designed to test the coadministration of Nigrocin-PN and antibiotics based on the references [27, 28]. The total volume for each well was 100 µl, which contained 98 µl of appropriate bacterial solution, 1 µl peptide solution, and 1 µl antibiotic solution. The designed concentration range was based on their MICs towards Nigrocin-PN and antibiotics, from 0.125 × MIC to 1 × MIC, and each combination has been repeated three times.

Parameter FICI (fractional inhibitory concentration indices) was used to evaluate the synergistic effect, and it was calculated according to the formula below [29]. A and B represent the MICs of drug A and drug B in the combination, while MIC_A_ and MIC_B_ represent the MIC values of the compounds alone. The FICI was interpreted as follows [30]: “synergy” (FICI ≤ 0.5), “no interaction” (0.5 < FICI ≤ 4), and “antagonism” (FICI > 4).

$$\mathrm{FICI}={\mathrm{FIC}}_{\mathrm{A}}+ {\mathrm{FIC}}_{\mathrm{B}}=\mathrm{A}/{\mathrm{MIC}}_{\mathrm{A}} +\mathrm{B}/{\mathrm{MIC}}_{\mathrm{B}}$$

### In vitro experimental evolution of resistance

Based on the results from the synergistic effect between Nigrocin-PN and antibiotics, *S. aureus* was chosen as the reference strain to study the evolution of resistance due to the significant synergy between Nigrocin-PN and ampicillin (*FICI* = 0.325). Four independent cultures were propagated in parallel with diluted drug background that was slightly lower than MICs. The drug concentrations for each group were ½ MIC Nigrocin-PN (2 µM), ¼ MIC ampicillin (0.25 µM), and ½ MIC Nigrocin-PN + 1/8 MIC ampicillin [31]. During the culturing, cell growth was measured by the OD_550nm_ to ensure the minimum absorbance over 0.2 to avoid population extinction [32]. When the OD values reached 0.3, each group was transferred to the new tube with fresh medium, with an inoculum ratio of 1:10. After about 20 cycles, bacteria in each group were subjected to MIC assay as described above in drug-free medium, and MICs of Nigrocin-PN and ampicillin were acquired.

### In vivo antimicrobial assay

BALB/c mice (male, 6–8-week old) which were purchased from Qinglongshan Laboratory Animal Company (Nanjing, China) were used to test peptides in vivo bio-ability. The animals were kept under SPF (specific-pathogen-free) conditions at 18 ~ 25 °C and 50 ~ 60% humidity, and all the procedures involving animals were approved by the Animal Care and Use Committee of Nanjing University of Chinese Medicine. Animals were stochastically divided into four groups: normal control group, drug-free control group, drug-exposure group, and positive group. Initially, 60 µl of inoculum of *K. pneumoniae* (3.13 × 10^7^ CFU/ml) was given nasally to mice in each group except the normal control group which was nasally given medium instead of bacteria. After 1 h of infection, peptides at 20.0 mg/kg and norfloxacin at 20.0 mg/kg were intraperitoneally injected [14, 33, 34]. While in the drug-free control group, the same volumes of saline were injected intraperitoneally instead. Mice were euthanized after 24 h of administration; then, the bilateral lung lavage was conducted. Eight-hundred microlitre of PBS was infused intratracheally and sucked back, and it was repeated three times. At last, approximately, 2 m µl of BALF (bronchoalveolar lavage fluid) was collected to detect numbers of bacteria colonies. Then, for the mouse in the same group suffered no procedures, the right middle lobe was fixed and stained with haematoxylin and eosin for pathologic observation; the right down lobe was used to measure the degree of oedema which was calculated by the ratio of wet weight/dry weight.

### Statistical analyses

Data are expressed as mean ± standard deviations (SD). One-way ANOVA was used to compare two groups in multigroup experiments. All the statistical analyses were performed by GraphPad Prism 7 (GraphPad Software, USA).

### Accession number

GenBank accession number of Nigrocin-PN is MT048670.1.

## Results

### “Shotgun” cloning of cDNA encoding a peptide precursor

**Fig. 1:**
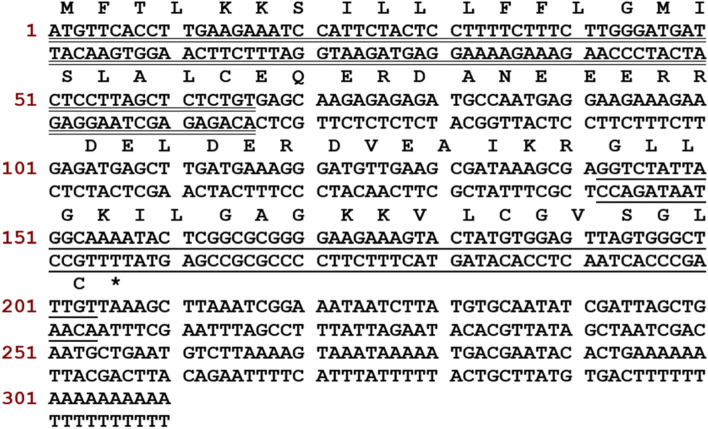
Nucleotide sequence and translated open-reading frame of the cDNA-encoding peptide precursor (Nigrocin-PN). The putative signal peptide is double underlined, followed by an acid space interval, and the mature peptide is single underlined, while the stop codon is indicated by an asterisk

The open-reading frame is comprised of 206 bp nucleotides (Fig. 1). To distinguish the putative signal peptide, both BLAST and the online signal-identification tool — SignalP 3.0 server (https://services.healthtech.dtu.dk/service.php?SignalP-3.0), were used. Alignment results were shown in Table 1. Comparison results revealed that the highly conserved N-terminal 22 amino acid residues encoded a putative signal peptide, and the most likely cleavage site was between positions 22 and 23. Between the signal peptide and the mature peptide, there was an amino acid residue-rich “spacer” region of 25 amino acid residues ended with − KR — which is a recognition site of classical endogenous propeptide convertases [35]. A putative mature peptide consisted of 21 amino acid residues (GLLGKILGAGKKVLCGVSGLC) and displayed a high consensus with other peptides from the nigrocin-2 family found putative mature peptide named Nigrocin-PN. According to alignment results, the putative mature peptide showed 85.7% identity with nigrocin-2 (GLLSKVLGVGKKVLCGVSGLC).

**Table 1:** Alignment of the putative signal peptide of *P. nigromaculatus* with those of other amphibian skin signal peptides representing the top ten identities based on BLAST

MFTLKKSILLLFFLGMISLALC	*Pelophylax nigromaculatus*	*E*-value
–––––––L–––––––––-S––	(1, 2, 3, 4, 5, 6, 7, 8, 9)	4e-12
–––––––L–––––––V––––-	(10)	4e-12

Dotting lines represent identical residues. 1, *Sylvirana maosonensis* (ALL26322.1). 2, *Amolops torrentis* (ADV36220.1). 3, *Amolops torrentis* (ADV36218.1). 4, *Amolops wuyiensis* (AIU99944.1). 5, *Amolops daiyunensis* (AIU99878.1). 6, *Amolops mantzorum* (ADM34271.1). 7, *Amolops granulosus* (ADM34228.1). 8, *Amolops granulosus* (ADM34227.1). 9, *Amolops torrentis* (ADV36217.1). 10, *Odorrana livida* (CAL25905.1). Accession numbers were given in brackets

### Purification and identification of Nigrocin-PN

**Fig. 2:**
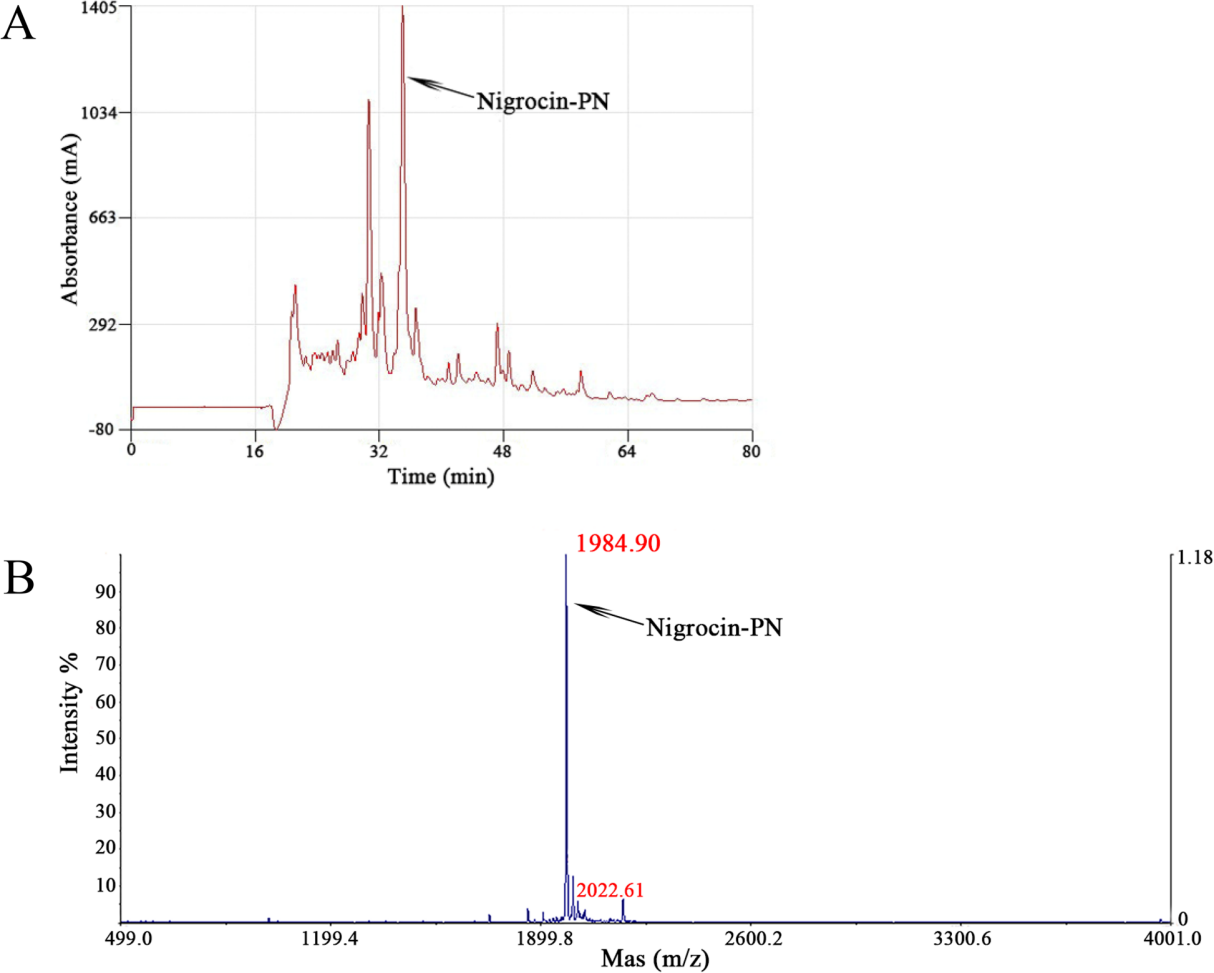
Purification of Nigrocin-PN. **A** is the RP-HPLC chromatogram of the crude synthetic peptide. The highest absorbance peak indicated by an arrow shows the position of the targeted peptide, x-axis shows the retention time, while the y-axis displays the relative absorbance. **B** is the MALDI-TOF mass spectrum of Nigrocin-PN, the x-axis is mass to charge ratio (m/z), and y-axis is the intensity of the substance. m/z 1984.91 indicated the singular protonated molecular mass, while m/z 2022.61 is identified as Nigrocin-PN with potassium adduct ion; no distinct peak at m/z 3986 reveals a high ratio of inner-molecular disulphide bond was formed

The synthetic peptide was subjected to the RP-HPLC, and the chromatogram was shown in Fig. 2. After the collection of the targeted fraction, MALDI-TOF was subsequently applied to identify the molecular weight of the peptide further. The major peptide ion was observed at m/z 1984.90, which is near the theoretical mass value regardless of the error.

### Secondary structure characterization of Nigrocin-PN and two other designed analogues

**Fig. 3:**
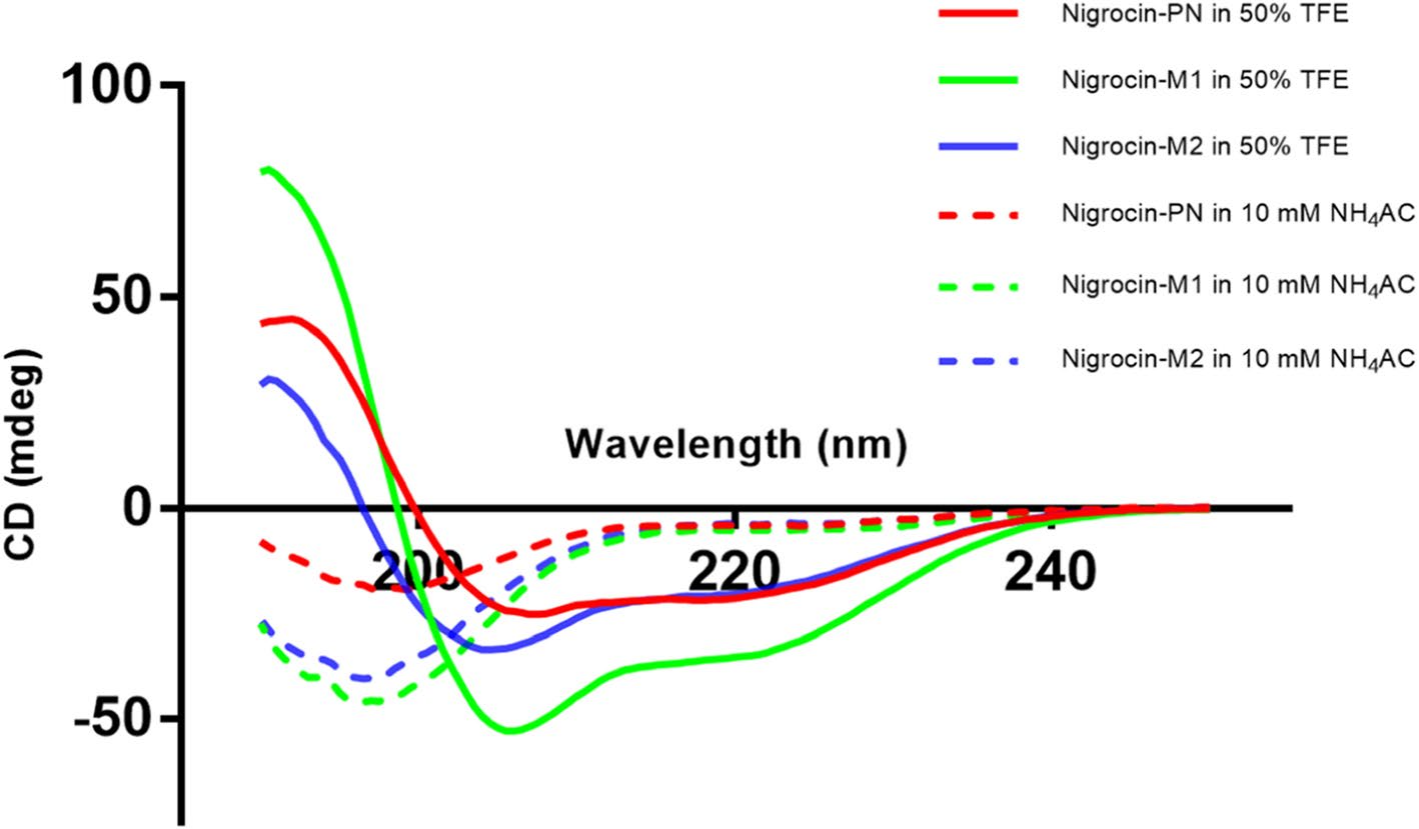
CD spectra of Nigrocin-PN (red), Nigrocin-M1 (green), and Nigrocin-M2 (blue) in different solutions. The CD spectra in 50% TFE/10 mM NH_4_AC solution and 10 mM NH_4_AC buffer at 20 °C are indicated by a solid and dotted line, respectively

All the peptides displayed random coil conformations in the aqueous environment (Fig. 3), whereas the proportion of α-helix structure was improved significantly when they were in 50% TFE. The α-helix of Nigrocin-PN in TFE environment was lower than its two modified analogues, which was basically due to the existence of the heptapeptide motif, and the disulphide bond prevented the further formation of α-helix. Nigrocin-M1 and truncated version Nigrocin-M2 formed a more complete α-helix structure in 50% TFE. The net charges of the peptide were not changed before and after the modification (Table 2).

**Table 2:** Estimated physiochemical properties and contents of the secondary structure of Nigrocin-PN, -M1, and -M2 in different solutions

	Hydrophobicity (H)	Hydrophobic moment (µH)	Net charge (Z)	α-helix (in NH_4_AC/in TFE)	β-sheet (in NH_4_AC/in TFE)	Random coil (in NH_4_AC/in TFE)
Nigrocin-PN	0.549	0.434	+ 3	0.03/0.73	0.20/0.03	0.77/0.24
Nigrocin-M1	0.558	0.427	+ 3	0.07/1.00	0.08/0.00	0.85/0.00
Nigrocin-M2	0.511	0.565	+ 3	0.01/0.99	0.01/0.00	0.98/0.01

### In vitro antimicrobial activities

The naturally occurring peptide Nigrocin-PN exhibited wide spectra antimicrobial abilities towards all tested microorganisms (Table 3). While Nigrocin-M1 showed less noticeable potency against some gram-negative bacteria (such as *E. faecalis* and *P. aeruginosa*, MIC values towards these pathogens were over 256 μM), with no compromised ability towards gram-positive strains though. The antimicrobial abilities of the truncated version Nigrocin-M2 decreased dramatically (MICs were almost over 512 μM), revealing the importance of “Rana box” for Nigrocin-2 family to express their bactericidal functions. Intriguingly, Nigrocin-M1 displayed enhanced inhibition abilities towards MRSA (B038 V1S1A, MIC of which was16 μM) and *S. constellatus* (B003 VISIT, MIC of which was 4 μM), which was twofold compared to the natural peptide.

**Table 3:** Antimicrobial activity of Nigrocin-PN and its derivatives against planktonic cells of gram-positive and gram-negative strains, as well as *C. albicans*

Bacterial strains	Nigrocin-PN (MIC/MBC) (µM)	Nigrocin-M1 (MIC/MBC) (µM)	Nigrocin-M2 (MIC/MBC) (µM)
*S. aureus* (NCTC 10,788)	4/8	4/8	512/512
*S. aureus* (ATCC 25,923)	16/64	16/32	512/ > 512
*E. coli* (NCTC 10,418)	8/8	8/16	128/128
*C. albicans* (NYCY 1467)	32/128	32/128	> 512/ > 512
*E. faecalis* (NCTC 12,697)	8/8	> 512/ > 512	> 512/ > 512
MRSA (NCTC 12,493)	16/16	16/16	> 512/ > 512
MRSA (B038 V1S1A)	32/64	8/32	> 512/ > 512
MRSA (B042 V2E1A)	32/64	16/32	> 512/ > 512
*S. constellatus* (B003 VISIT)	8/8	4/4	256/256
*K. pneumoniae* (ATCC 43,816)	16/32	16/16	256/256
*K. pneumoniae* (ATCC 13,883)	16/64	8/32	512/ > 512
*P. aeruginosa* (ATCC 27,853)	64/64	512/512	512/ > 512
*P. aeruginosa* (ATCC 9097)	64/128	256/512	512/ > 512
*P. aeruginosa* (B004 V2S2B)	16/32	> 512/ > 512	> 512/ > 512

Antibiofilm profiles of the peptides were summarised in Table 4. Consistent with antimicrobial assays, the efficacy of Nigrocin-M2 against sessile cells of *S. aureus* and MRSA in biofilm was prominently reduced (both MBIC and MBEC were over 256 μM) compared to the natural peptide and the “L”-substituted version. MBIC of Nigrocin-M1 was similar to Nigrocin-PN on both strains, while the MBEC of Nigrocin-PN (4 μM) was fourfold compared to Nigrocin-M1 (16 μM) on MRSA.

**Table 4:** Antibiofilm activity of Nigrocin-PN and its derivatives against *S. aureus* and MRSA

Bacterial strains	Nigrocin-PN (MBIC/MBEC) (µM)	Nigrocin-M1 (MBIC/MBEC) (µM)	Nigrocin-M2 (MBIC/MBEC) (µM)
*S. aureus* (NCTC 10,788)	8/8	8/8	256/512
MRSA (NCTC 12,493)	16/4	16/16	> 512/ > 512

### Antimicrobial mechanisms probing

**Fig. 4:**
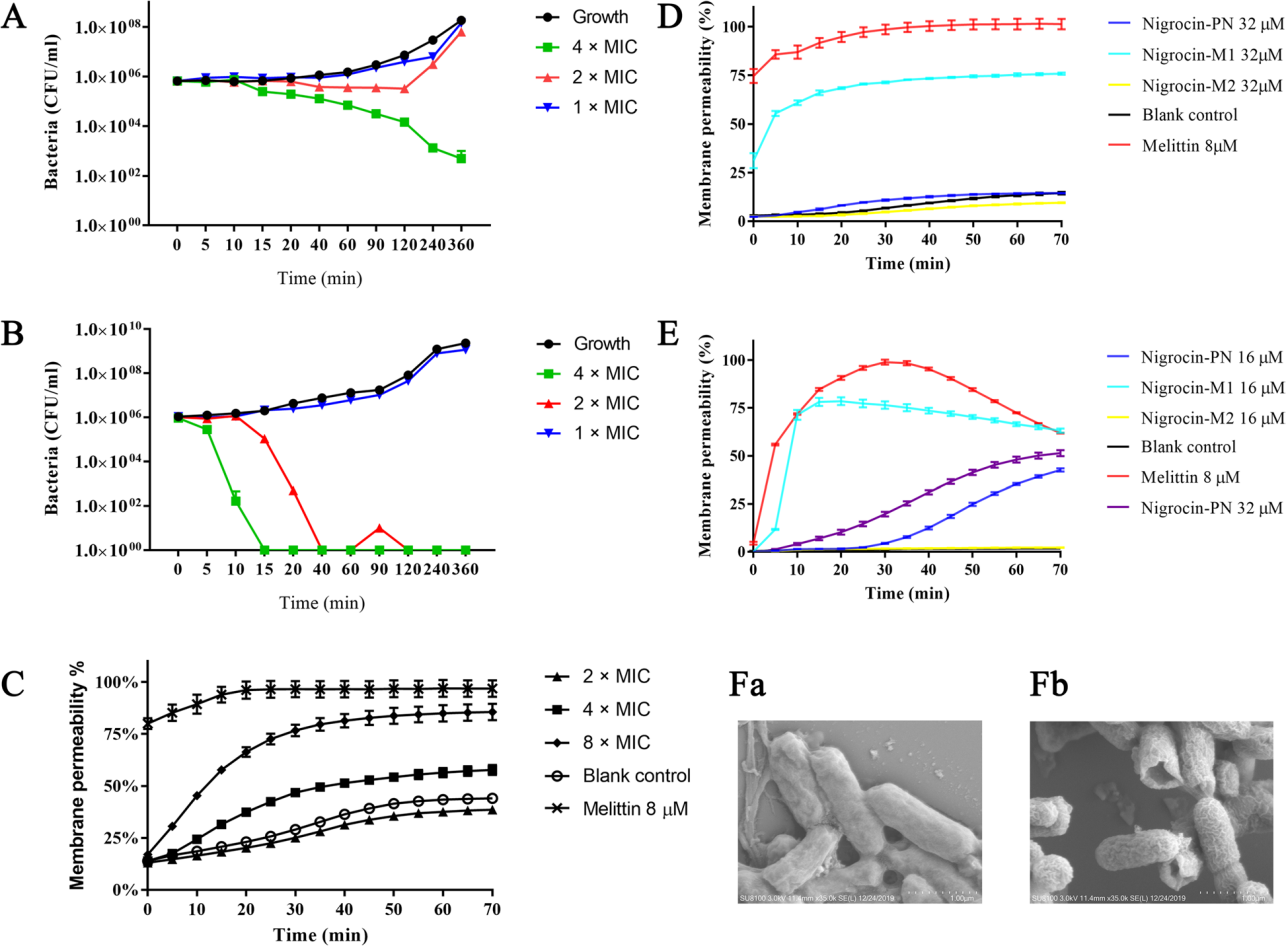
Time-killing curves for Nigrocin-PN on *S. aureus* (**A**) and *E. coli* (**B**); the concentration of Nigrocin-PN added was fourfold, twofold, and onefold of its MIC. Dynamic membrane permeability curves of Nigrocin-PN at different concentrations towards *S. aureus* (**C**). Permeability curves of Nigrocin-PN, Nigrocin-M1, and Nigrocin-M2 on MRSA (NCTC 12,493) (**D**) and on *E. coli* (**E**), the determined concentrations for Nigrocin-PN and Nigrocin-M1 were performed at twofold of their MICs, Nigrocin-M2 kept the same concentration in each group for better comparison, and 8 µM melittin in PBS was used as a positive control. The error bar represents the standard error for three repeats. Fa and Fb were *K. pneumoniae* (ATCC 13,883) treated with 0 and 16 µM Nigrocin-PN under 24 h of incubation, respectively

The inhibition efficacy for Nigrocin-PN at MIC was not as significant as the concentration at 2 × MIC and 4 × MIC, revealing the enhanced bacteria inhibition as the concentration improved (Fig. 4 A and B). At twofold of MIC towards *E. coli*, time-killing assay indicated no distinct survival at around 40 min, whereas the membrane permeability rate was about 12.5% at this time, indicating the possible bactericidal mechanism by non-membrane destruction at relatively lower concentration (Fig. 4 B and E), because membrane destruction was not sufficient to explain 100% bacterial death. However, when the given concentration was over 4 × MIC, the membrane rapture rate increased sharply with the extension of time (Fig. 4C). Based on the data, the possible antibacterial mechanisms for Nigrocin-PN could be bilateral; it could inhibit bacterial reproduction by mediating non-membrane destruction at low concentrations, while directly destroying bacterial cell membrane at high concentrations. Nevertheless, prolonged incubation time between Nigrocin-PN and bacteria could inevitably destroy the bacterial membrane and cause bacterial death (Fig. 4 Fa and b).

In MRSA and *E.coli* conditions, as shown in Fig. 4 D and E, both Nigrocin-PN and Nigrocin-M1 were tested at 2 × MIC. Nigrocin-M1 showed a rapid disruption of membrane trend, while Nigrocin-PN was not differentiated from the blank control at the beginning. Nevertheless, they kept the same MIC value for MRSA (NCTC12493) and *E. coli* (NCTC 10,418). The possible explanation for this phenomenon would be the effects of the C-terminus “Rana box.” Due to the absence of the intramolecular disulphide bond, Nigrocin-M1 could quickly target the negatively charged peptidoglycan and teichoic in MRSA and bind to LPS in *E. coli* and then further initiate the membrane disruption. However, due to the complexity of MoAs (mode of actions), further experimental confirmation of the mechanism is required, while the truncated analogue Nigrocin-M2 lost the membrane destruction ability (Fig. 4 D and E) and showed no statistical difference compared with the blank control. The presence or absence of “Rana box” is directly related to their antibacterial activity.

Thus, the existence of “Rana box” helped to keep the peptide’s antibacterial ability and endowed unique antibacterial mechanisms at low concentrations.

### Cytotoxicity and anticancer assessment

**Fig. 5:**
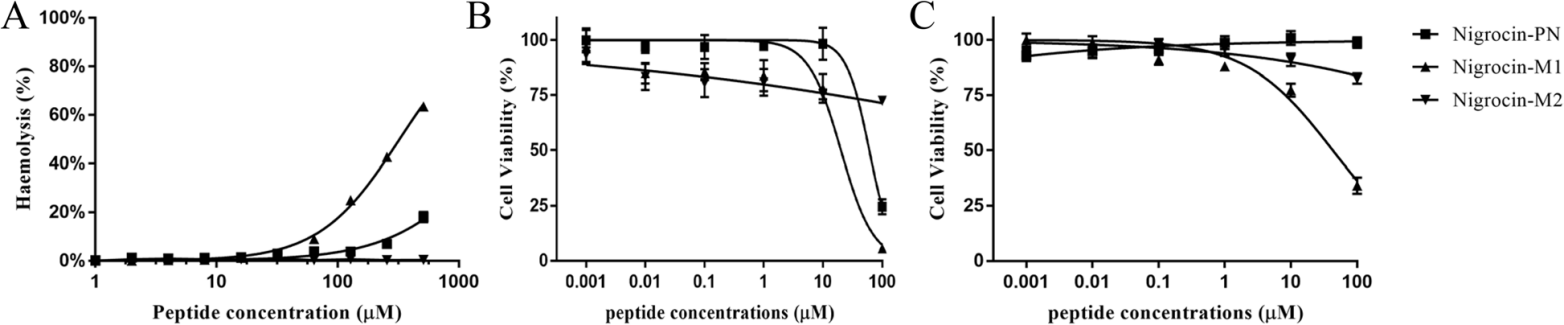
**A** Relative haemolysis of Nigrocin-PN and its derivates tested on defibrated horse blood cells, 1% Triton X-100 was set as a positive control. Cytotoxicity assessment on human keratinocyte cell line HaCaT (**B**) and on human microvessel endothelial cell line HMEC-1 (**C**). The error bar represents the standard error for five repeats

As shown in Fig. 5A, compared to Nigrocin-PN and Nigrocin-M2, the damage of the disulphide bond improved the haemolysis index significantly, while the total deletion of “Rana box” decreased the haemolysis, especially when the concentration was over 100 µM. Furthermore, the deletion of “Rana box” seemed to affect the peptide’s overall biological activity, since Nigrocin-M2 exhibited no significant cell cytotoxicity, mirroring its weak antimicrobial activities (Fig. 5 B and C and Table 3).

Both Nigrocin-PN and Nigrocin-M1 presented minor anticancer abilities. Nigrocin-PN showed no antiproliferation activity towards MCF-7. The anticancer ability of Nigrocin-M1 was enhanced among the tested cell lines except for H157, while there was no detectable IC_50_ for Nigrocin-M2 (Table 5).

**Table 5:** Anticancer activity of Nigrocin-PN and its derivates against several cancer cell lines

	IC_50_ (µM)		
	Nigrocin-PN (µM)	Nigrocin-M1 (µM)	Nigrocin-M2 (µM)
H157	18.04	24.00	ND
PC-3	53.03	31.27	ND
U-251MG	73.65	25.06	ND
MCF-7	ND	24.60	ND

*ND*, Not determined, IC_50_ was over 100 µM

### Synergistic effect between Nigrocin-PN and antibiotics

The synergy effect between Nigrocin-PN and ampicillin to treat *S. aureus* was observed, with FICI at 0.375. Non-antagonism effect was observed among all the studies. For Nigrocin-PN, the most synergy effect with antibiotics was among gram-positive strains, as it showed a lower combination efficacy towards *E. coli* (Table 6). Thus, to better understand the combination benefits between Nigrocin-PN and antibiotics, the drug pair Nigrocin-PN–ampicillin was chosen in the evolution resistance study.

**Table 6:** The combination effects of Nigrocin-PN with different antibiotics treated on several microbial strains

Strains	Norfloxacin (lowest FICI)	Ampicillin (lowest FICI)	Vancomycin (lowest FICI)	Gentamicin (lowest FICI) c
*S. aureus* (NCTC 10,788)	0.625 (NI^a^)	0.375 (S^b^)	0.625 (NI)	0.620 (NI)
MRSA (NCTC 12,493)	\^c^	\	1.125 (NI)	0.500 (S)
*E. coli* (NCTC 10,418)	0.625 (NI)	\	\	0.750 (NI)
*C. albicans* (NYCY 1467)	0.500 (S)	\	0.375 (S)	\

“NI^a^” represents “no interaction.”

“S^b^” represents “synergy.”

“\^c^” represents “not studied.” Only the MICs of antibiotics fell between 64 and 0.125 µM were chosen to study the combination effect

After only 20 generations, bacterial populations evolving in the presence of a single antibiotic (ampicillin) reached eightfold increases in ampicillin MIC level relative to its ancestor. In contrast, Nigrocin-PN and ampicillin co-administrated significantly slowed down the evolution of antibiotic resistance, since the MIC of ampicillin was only twofold of its ancestor, although this process happened under the sacrifice of minor resistance for bacterium towards Nigrocin-PN. The susceptibilities for bacterium passaged only in the background of Nigrocin-PN were unchanged towards the peptide and the antibiotic, which were 16 and 0.5 µM, respectively (Table 7).

**Table 7:** The MIC changes for Nigrocin-PN and ampicillin in different processed *S. aureus* strains

Different passage process groups	MIC (µM)	
	Nigrocin-PN	Ampicillin
Group A strain	16	0.5
Group B strain	16	4.0
Group C strain	16	0.5
Group D strain	32	1.0

Group A strain: *S. aureus* was passaged under drug-free background

Group B strain: *S. aureus* was passaged under ¼ MIC ampicillin stress

Group C strain: *S. aureus* was passaged under ½ MIC Nigrocin-PN stress

Group D strain: *S. aureus* was passaged under the combination of ½ MIC Nigrocin-PN and 1/8 MIC ampicillin

Thus, the concurrent administration of Nigrocin-PN and ampicillin could not only show synergistic effect but delayed the acquisition of resistance for *S. aureus* compared to using ampicillin alone.

### In vivo antimicrobial assay

**Fig. 6:**
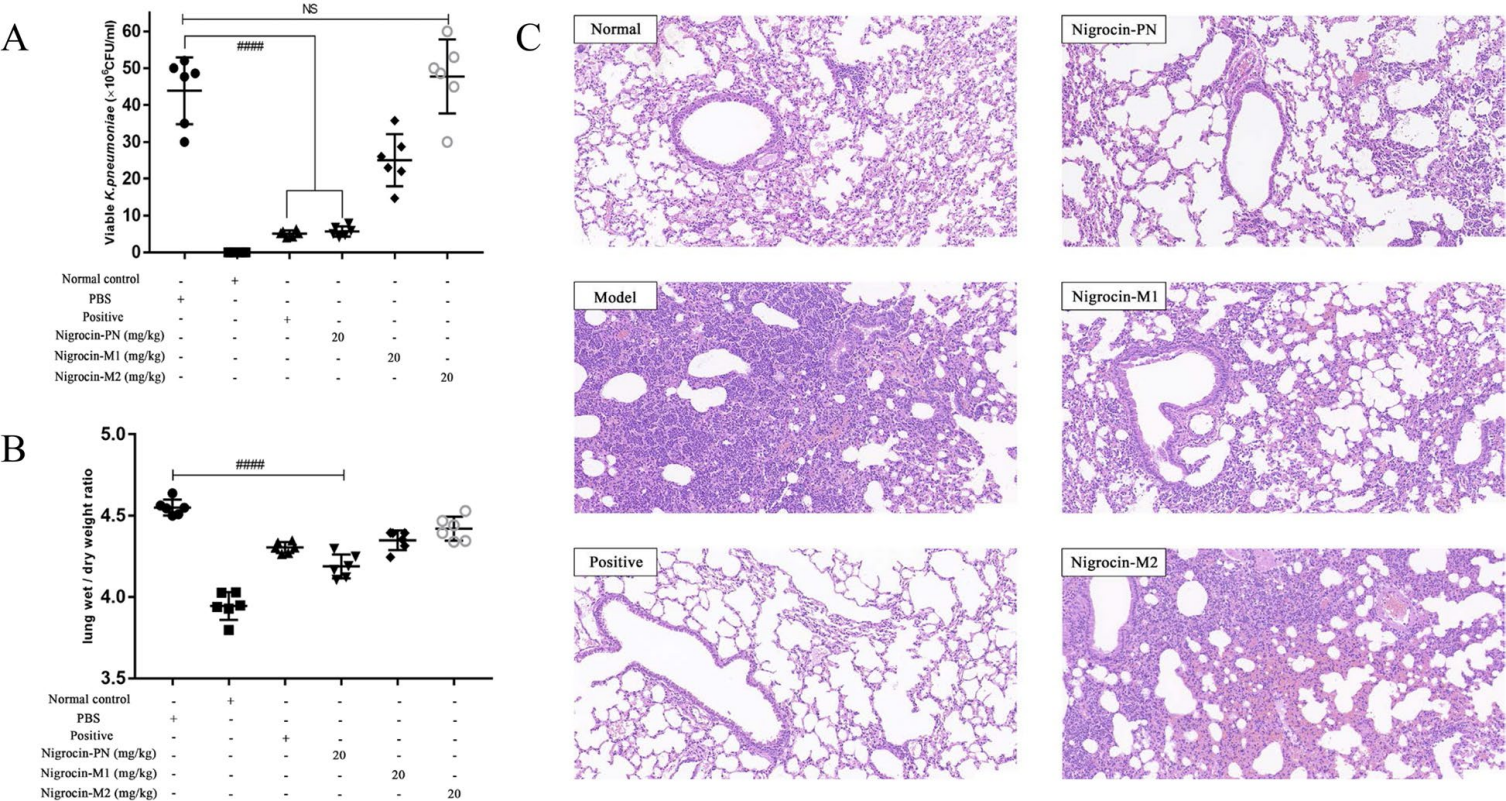
Efficacy of peptides in the *K. pneumoniae*-induced pneumonia mice model. The number of *K. pneumoniae* CFU in the BALF was calculated from the number of colonies growing on MHA plates (**A**). Effects of Nigrocin-PN and derivates on lung inflammation caused by *K. pneumoniae* were indicated by wet/dry ratio (**B**). The right middle lobe was further fixed and stained with haematoxylin and eosin, the magnification is 20 × , and the bar length represents 100 µm (**C**). ####*P* < 0.0001 versus the drug-free control group (PBS); NS means not statistically significant

Compared to the two modified peptides, Nigrocin-PN exhibited prominent in vivo antimicrobial ability against *K. pneumoniae*. As shown in Fig. 6A, Nigrocin-PN could ameliorate the lung infection at 20.0 mg/kg, as evidenced by reduced viable bacteria in BALF of infected mice. Moreover, pathological changes were indicated by the wet/dry ratio of lungs and HE staining (Fig. 6 B and C), which revealed Nigrocin-PN markedly mitigated pulmonary inflammation and oedema caused by *K. pneumoniae*. Meanwhile, Nigrocin-M1 showed a similar pattern with relatively weaker potency.

## Discussion

To date, antimicrobial peptide families discovered in *Pelophylax nigromaculatus* include pelophylaxin, temporin, nigrocin, and esculetin. Nigrocins are mainly distributed in genera *Odorrana* and *Pelophylax*. Thus, there are only several nigrocins discovered. Among them, Nigrocin-PN is the most prominent broad-spectrum antimicrobial peptide with the lowest haemolysis index.

Based on in vivo and in vitro studies above, the bactericidal activity is highly speculated to link with the seven amino acids formed “Rana box” (C-terminal loop). The same pattern was also observed in thanatin (a host defence peptide from an insect *P. maculiventris*), which showed improved ability by deleting threonine from eight amino acids formed C-terminus loop to seven amino acids formed loop and weakened ability by extending the length of the loop; furthermore, two cysteine residues replaced analogue was found to be largely inactive [36, 37]. In Nigrocin-M1, when two cysteines were replaced by leucine, deceased bactericidal abilities towards gram-negative strains were observed; it is presumably because the lack of C-terminus loop makes it easier to get aggregated and become neutralised in LPS of the outer leaflet of the outer membrane of gram-negative bacteria, while the existence of C-terminus loop prevents self-aggregation and strengthens the membrane disruption effects [38, 39].

Many studies attribute the antibacterial abilities to the peptides’ net charge. However, the charge is not the only thing that determines the antibacterial efficiency. For instance, three involved peptides hold the same net charges (Table 2) but showed varied attributes. What’s more, they have different cytotoxicity profiles. Nigrocin-PN showed prominent functions towards pathogens, though it showed limited haemolysis even under the highest test concentration of 512 μM. For Nigrocin-M1, improved cytotoxicity was observed. Combining with CD data, net charge and α-helix may not be as important as C-terminus loop herein, for which may interact with some motifs such as LPS on bacteria which are lacking on normal cells [7], and this reflects the selectivity and safety of the action of Nigrocin-PN.

SYTOX green and morphology study revealed that the potential bactericidal mechanism was membrane destructing, although the detailed molecular level interactions still need to be investigated. However, this explains why Nigrocin-PN shows some degrees of synergistic effects with antibiotics. Most antibiotics target the bacterial cell wall, DNA, or ribosomes; since Nigrocin-PN could improve bacterial membrane permeability, it may facilitate antibiotics’ cytoplasmic membrane translocation and thereby strengthen their functions.

## Conclusions

A newly identified bioactive peptide derived from *Pelophylax nigromaculatus* was discovered, the sequence of which was GLLGKILGAGKKVLCGVSGLC. Structurally, intramolecular disulphide bond helped to keep the peptide bioactivity and decrease cytotoxicity to normal cell lines. Furthermore, the discovered peptide showed synergistic effects with commonly used antibiotics, especially with ampicillin fight against *S. aureus*. The potential bactericidal mechanisms involved membrane destruction.

## Data Availability

Not applicable.

## Abbreviations

AMP(s): Antimicrobial peptide(s)
HDPs: Host defence peptides
RACE: Rapid amplification of cDNA ends
NUP: Nested universal primer
CD: Circular dichroism
MIC: Minimal inhibitory concentration
MBC: Minimal bactericidal concentration
MBIC: Minimal biofilm inhibitory concentration
MBEC: Minimal biofilm eradication concentration
FICI: Fractional inhibitory concentration indices
